# Attitudes Toward the Copper IUD in Sweden: A Survey Study

**DOI:** 10.3389/fgwh.2022.920298

**Published:** 2022-07-08

**Authors:** Maria Wemrell, Lena Gunnarsson

**Affiliations:** ^1^Department of Gender Studies, Lund University, Lund, Sweden; ^2^Unit for Social Epidemiology, Department of Clinical Sciences, Lund University, Malmö, Sweden; ^3^School of Humanities, Education and Social Sciences, Örebro University, Örebro, Sweden

**Keywords:** copper IUD, attitudes, survey, Sweden, women's health

## Abstract

**Background:**

While the efficacy and safety of the contraceptive copper intrauterine device (IUD) have been affirmed, alongside its importance for the prevention of unintended pregnancies, some studies have pointed to negative attitudes toward the device. In recent years, social media communication about it has included claims about systemic side effects, unsubstantiated by medical authorities. Research from the Swedish context is sparse. This study investigates attitudes toward the copper IUD and any correlations between negative attitudes toward or experiences of the device, and (1) sociodemographic characteristics, (2) the evaluation of the reliability of different sources of information, and (3) trust in healthcare and other societal institutions.

**Methods:**

A survey was distributed online to adult women in Sweden (*n* = 2,000). Aside from descriptive statistics, associations between negative attitudes toward or experiences of the copper IUD and sociodemographic and other variables were calculated using logistic regressions and expressed as odds ratios (ORs) with 95% confidence intervals (95% CIs). Open survey responses (*n* = 650) were analyzed thematically.

**Results:**

While many reported positive attitudes toward and experiences of the IUD, 34.7% of all respondents reported negative attitudes and 45.4% of users reported negative experiences. Negative attitudes were strongly correlated with negative experiences. Negative attitudes and experiences were associated with low income, but no conclusive associations were identified with other socioeconomic variables. Negative attitudes and experiences were associated with lower levels of confidence in and satisfaction with healthcare, as well as lower self-assessed access and ability to assess the origin and reliability of information about the IUD. In open responses, negative comments were prevalent and included references to both common and unestablished perceived side-effects. Respondents pointed to problematic aspects of information and knowledge about the copper IUD and called for improved healthcare communication and updated research.

**Conclusion:**

Healthcare provider communication about the copper IUD should promote reproductive autonomy and trust by providing clear information about potential side effects and being open to discuss women's experiences and concerns. Further research on copper IUD dissatisfaction and ways in which health professionals do and may best respond to it is needed.

## Introduction

The importance of long-acting reversible contraceptive methods (LARC), including copper intrauterine devices (IUDs), for the prevention of unintended pregnancies and their costs (1) is emphasized internationally (2) and in Sweden (3, 4). The efficacy and safety of the copper IUD have been affirmed (5–9), and in Sweden, medical guidelines point it out as a suitable first-hand choice to most women desiring a LARC method (10). In Sweden, where rates of unintended pregnancy are relatively high (11, 12), the use of LARC, including the copper IUD, increased between 2010 and 2017 (11, 13).

While research points to high levels of satisfaction with LARC methods including the copper IUD (6, 14, 15), studies have also indicated negative attitudes toward the copper IUD in countries including Sweden (14, 16–21). Negative views on the copper IUD have often been associated with communication in social networks, including online social media, where negative commentaries on IUDs have been found to be prevalent (14, 16, 22–27), although positive views are also communicated (23, 25). Meanwhile, the strong importance of social networks for contraceptive choice and use has been observed in countries including Sweden (28, 29). Studies indicate that women often consult both “official” and “unofficial” sources regarding the copper IUD (16, 22), and that some women consider accounts relayed through social contacts or from individuals with personal experience to be more reliable than information from healthcare providers (22, 30–32).

Today, the seeking and sharing of health-related information via social media have raised widespread concern about the spread of misinformation to the potential detriment of individual and public health (33, 34). This has been related to the contemporary contestation of expert knowledge (35) in what has been called a “post-truth” era (36), strongly actualized in recent years not least in relation to vaccine hesitancy (37–39). Such contestation of established expertise has often been understood in terms of misunderstanding of science (40), public knowledge deficits [(40, 41), or misinformation (33, 34). Accordingly, studies observing negative attitudes toward the copper IUD, not least as communicated online and through other social networks, have emphasized the importance of countering misinformation (22, 30, 32, 42). Negative or hesitant views on medical matters, which misalign with established expertise, have furthermore been associated with a lack of trust not only in the medical products themselves but in organizations producing and administering them and in societal institutions at large (43, 44). Such negative or hesitant views have been found to be unevenly distributed between sociodemographic groups, with stronger hesitancy in the less privileged strata ((45, 46)). Meanwhile, the establishment of trust has been noted by women as being a fundamental precondition for clear communication with healthcare providers about contraceptive methods (47). Mistrust in healthcare providers, alongside concerns with the quality of healthcare, has also been tied to resistance to or discontinuation of IUD use (14).

In recent years, some online communication about the copper IUD has centered on claims about systemic side effects, encompassing both physical and mental symptoms attributed to the release of copper in the body and the inflammation caused by the IUD. Similar claims have been noted in passing in previous research (21, 25, 48, 49). In Sweden, a social media group focused on this issue currently includes around 8,500 members (17), and it is unknown whether such notions are more widely spread in the population. In line with the concern about misinformation noted above, these claims have been referred to as “alternative facts” spreading through social media, comparable to, for example, notions of vaccine side effects or protective effects of female genital mutilation (50).

At the same time, women's health movements and feminist research have long placed focus on ambivalent or negative effects of contraceptive methods on the health and wellbeing of women (51–54). While recognizing the central importance of preventing unwanted pregnancies, not least in the interest of women's empowerment, such movements and researchers have, sometimes invoking the concepts of reproductive autonomy, justice, and rights, pointed to side effects and coercive practices associated with contraceptives, including copper IUDs (55–57), not least in the Global South (53, 58, 59) and among less privileged groups in the North (55, 60). In some cases, such movements have contributed toward acknowledgment of previously unrecognized side effects and to litigation and product recall (56, 57, 61). Research has furthermore pointed to women's experienced side effects of contraceptive methods not being highly prioritized, and to women finding it difficult to voice their concerns about such side effects to and receive sufficient information about them from medical professionals (31, 62–64).

Information-sharing, communication, and peer support via social media can have positive effects on knowledge about and involvement in health (65–67), potentially alleviating socioeconomic inequalities in health (65) and decreasing barriers to information exchange among more marginalized groups in society (68, 69). Such positive potential effects exist alongside, and in possible tension with, detrimental effects of the spreading of misinformation.

Against this background, the present study aims to investigate attitudes toward the copper IUD among adult women in Sweden. Attention is directed at the evaluation of the reliability of different sources of knowledge and at any mentions of unestablished, systemic side effects, as well as at any correlations between negative attitudes toward or experiences of the IUD and sociodemographic characteristics or levels of trust in sources of information. This is in line with research highlighting the need to study factors influencing women's contraceptive choices, including women's experiences of and attitudes toward contraceptives (16, 21, 62), not least due to the noted importance of informal social communication for such choices (70).

## Materials and Methods

This survey study, conducted online in collaboration with the market research company Kantar Sifo (www.kantarsifo.se), targeted adult women (18–55 years, *n* = 2,000) across Sweden. The respondents were randomly selected from Kantar Sifo's web panel, which includes approximately 100,000 active panelists recruited through nationally representative random selection (71). The sample size (*n* = 2,000) was intended to allow for some sub-group analysis.

The questionnaire was developed by the researchers in cooperation with Kantar Sifo and encompassed previously used items (72) alongside others constructed for the current study. The questions inquired about past, present, and likely future use of a copper IUD and, drawing on the Health Belief Model (73), about perceived benefits and risks of the device. Participants were also asked to evaluate the credibility of various sources of information about the copper IUD and to assess their general trust in different societal institutions. Sociodemographic information, apart from that already assembled by Kantar Sifo, was gathered. Most questions were closed, enabling quantitative analysis, while two open questions enabled the participants to formulate written responses. Alongside attitudes toward the copper IUD, addressed in this article, the questionnaire also posed similar questions about HPV vaccination (37). The survey, excluding the questions concerning HPV vaccination, can be found in the Appendix.

The response rate was 37%, which is in line with the average response rates of Kantar Sifo web panel surveys. Results were weighted with regard to age and region according to national weights provided by Statistics Sweden to improve representativity. Kantar Sifo's quality control, including response time and straight-liner analyses (74), deemed the data to be of good quality. The survey responses delivered to the research group were anonymized.

The project was approved by the Swedish Ethical Review Authority (no. 2019-03017).

### Data Analysis

Alongside descriptive statistics, we used multiple logistic regressions to measure any associations between negative attitudes toward or reported negative experiences of the copper IUD and sociodemographic and other variables. Associations were expressed as odds ratios (ORs) with 95% confidence intervals (95% CIs). All statistical analyses were conducted using IBM SPSS Version 27.0 (SPSS Inc., Chicago, IL, USA). Open-survey responses were analyzed thematically (75), i.e., divided into themes and categories, using NVivo.

### Assessment of Variables

The regression analyses were based on four variables pertaining to attitudes toward and experiences of the copper IUD. Primarily, a variable measuring attitudes toward the IUD was based on four survey questions, drawing on the Health Belief Model (73), asking about the perceived benefits and risks of the copper IUD. Those reporting weak or no agreement with one or more of the statements (*The copper IUD fills an important function in preventing unwanted pregnancy*; *The risks and side effects of the copper IUD are uncommon*; *The risks and side effects of the copper IUD are mild*; *The benefits of the copper IUD outweigh its potential risks*) were distinguished from those reporting fairly strong or strong agreement, or being unsure, in the dichotomized variable indicating attitude toward the copper IUD (*negative* vs. *positive or unsure*).

Secondly, the respondents were asked about the present or past use of a copper IUD. Those who reported no such use were asked if they would likely use one in the future, the response options being “*yes, it is likely*,” “*I do not know*,” “*no, because I don't need a contraceptive*,” “*no, because I prefer another contraceptive*,” or “*no, because I do not want to use a copper IUD*.” Those choosing the latter option, that they did not want to use a copper IUD, were distinguished from those responding anything else in a dichotomized variable indicating an unwillingness to use a copper IUD (*do not want to use a copper IUD* vs. *other*). Age-related factors likely affected the responses, as future use of a contraceptive method is more improbable in the oldest age group and younger respondents may lean toward a preference for another contraceptive method (76). However, as our interest here lies in expressions of unwillingness to use the IUD, chosen over and above other response options, we chose to include all age groups in the analysis.

Furthermore, respondents reporting using or having used a copper IUD were asked about whether their experiences were very or fairly positive, very or fairly negative, or if they were unsure. Those reporting very or fairly negative experiences were distinguished from those reporting positive experiences or being unsure in the dichotomized variable indicating experiences of the copper IUD (*negative* vs. *positive or unsure*).

Finally, a variable was constructed on the basis of two open questions included in the survey. The first of these questions was posed to participants responding that they did not want to use a copper IUD in the future and inquired about the reason for this. The second one, given to all the participants, invited any additional comments regarding the copper IUD. A number of the responses referred to claimed unestablished systemic side effects of the IUD, in many but not all cases linked to the release of copper in the body. No such perceived side effects were mentioned in the survey itself, or the accompanying information text. Participants making such references were distinguished from all others (*reference to unestablished systemic side effects* vs. *no such references*).

The other variables will be described in association with the results of each analysis.

## Results

Characteristics of the study population can be seen in Table 1.

**Table 1:** Unweighted study population characteristics and distribution of negative attitudes toward the copper IUD between sociodemographic groups.

**Attitude toward the IUD**	**Total**	**Negative**	**Positive/Unsure**
	***n* (%)**	***n* (%)**	***n* (%)**
Women	1,998 (99.9)	649 (32.5)	1,349 (67.5)
Other gender identity (with womb)	2 (0.1)	0 (0.0)	2 (100.0)
18–29 years	422 (21.1)	176 (41.7)	246 (58.3)
30–49 years	1,074 (53.7)	356 (33.1)	718 (66.9)
50–64 years	504 (25.2)	117 (23.2)	387 (76.8)
High education	1,466 (73.3)	472 (32.2)	994 (67.8)
Low education	530 (26.5)	176 (33.2)	354 (66.8)
High income	361 (18.1)	97 (26.9)	264 (73.1)
Medium income	999 (50.0)	305 (30.5)	694 (69.5)
Low income	502 (25.1)	212 (42.2)	290 (57.8)
Born in Sweden	1,923 (96.2)	624 (32.4)	1,299 (67.6)
Born elsewhere	75 (3.8)	24 (32.0)	51 (68.0)
Children	1,218 (60.9)	380 (31.2)	838 (68.8)
No children	513 (39.1)	269 (34.4)	513 (65.6)

### Attitudes Toward the Copper IUD

In response to questions about the perceived benefits and risks of the copper IUD (Table 2), a large majority (82.9%) reported agreeing, strongly or fairly strongly, that the device fills an important function in preventing unwanted pregnancy. A smaller share agreed, strongly or fairly strongly, that the risks and side effects of the copper IUD are uncommon (35.1%) and mild (35.2%). Around half (47.8%) agreed, strongly or fairly strongly, that the benefits of the copper IUD outweigh its potential risks. To the latter three questions, 17.3–25.8% reported agreeing less or not at all, while just over one-third (34.5–38.8%) responded being unsure. In the conflated variable noted above, used to measure attitudes toward the copper IUD (*negative* vs. *positive or unsure*), those indicating a negative attitude comprised 34.7% (*n* = 695) of the respondents.

**Table 2:** Perceived risks and benefits of the copper IUD.

	**Agree strongly *n* (%)**	**Agree to fairly high degree *n* (%)**	**Agree to fairly low degree *n* (%)**	**Disagree *n* (%)**	**Unsure *n* (%)**
The copper IUD fills an important function in preventing unwanted pregnancy	923 (46.1)	735 (36.8)	87 (4.4)	18 (0.9)	229 (11.4)
The risks and side effects of the copper IUD are uncommon	191 (9.5)	511 (25.6)	380 (19.0)	136 (6.8)	770 (38.5)
The risks and side effects of the copper IUD are mild	157 (7.8)	548 (27.4)	368 (18.4)	142 (7.1)	776 (38.8)
The benefits of the copper IUD outweigh its potential risks	355 (17.8)	600 (30.0)	228 (11.4)	119 (5.9)	690 (34.5)

To the question about past or present use of a copper IUD, 79.7% (*n* = 1,594) of the respondents indicated no such use. These respondents were asked if they would likely use a copper IUD in the future. The option “*no, because I do not want to use a copper IUD*,” given alongside the others noted above was chosen by 25.2% (*n* = 347) of these, indicating an unwillingness to use an IUD (*do not want to use a copper IUD* vs. *other*).

To the same question, 19.9% (*n* = 397) of the participants reported that they were using (5.1%, *n* = 102) or had been using (14.8%, *n* = 295) a copper IUD. The share of women aged 18–49 years reporting current copper IUD use was 4.7% (*n* = 76). Of all the current or past users, 54.7% (*n* = 217) reported having had very or fairly positive experiences (53.5%) or that they were unsure (1.2%). The remaining 45.4% (*n* = 180) reported having had very or fairly negative experiences, thus indicating negative experiences of the copper IUD (*negative* vs. *positive or unsure*).

In response to the survey's two open questions, and as described in more detail below, 42 participants (2.1%) referred to unestablished systemic side effects.

A negative attitude toward the copper IUD was, unsurprisingly, associated with unwillingness toward future use (OR 3.81 [95% CI 2.95–4.91]) and, strongly so, with negative experiences of the copper IUD (OR 15.66 [95% CI 9.53–25.76]). It was also associated with making references to systemic side effects (OR 5.86 [95% CI 2.99–11.48]) (Table 3).

**Table 3:** Associations between negative attitudes toward and negative experience of, unwillingness to use, and having made reference to copper-related or systemic side effect of the copper IUD, expressed as odds ratios (ORs) with 95% confidence intervals (CIs).

	**Negative attitude OR (95% CI)**
Unwillingness to use	3.81 (2.95–4.91)
Negative experiences	15.66 (9.53–25.76)
Reference to copper/systemic side effects	5.86 (2.99–11.48)

### Sociodemographic Factors

The unweighted distribution of negative attitudes toward the copper IUD across sociodemographic groups in the study population is shown in Table 1.

The education variable was defined as having a tertiary education or not (*high* vs. *low*). The income variable conflated categories used by Kantar Sifo and distinguished between those with a personal monthly income of SEK < 24,999 (*low*), SEK 25,000–41,999 (*medium*), and SEK >41,999 (*high*) (SEK 10,000=USD ~1,000). The age variable divided the participants into three groups (18–29; 30–49; 50–64 years). The country of birth variable distinguished between those born in Sweden or another country (*Sweden* vs. *elsewhere*). The parenthood variable distinguished between multiparous and nulliparous women (*children* vs. *no children*).

Any correlations between these socioeconomic variables and negative attitudes toward, negative experiences of, unwillingness to use, and references to systemic side effects of the IUD were measured individually, and when more than one variable showed a conclusive association, these were entered jointly in a new regression model. The remaining conclusive associations are displayed in Table 4.

**Table 4:** Associations between sociodemographic characteristics and negative attitudes toward, negative experience of, unwillingness to use or having made reference to copper-related or systemic side effects of the copper IUD, expressed as odds ratios (ORs) with 95% confidence intervals (CIs).

	**Negative attitude OR (95% CI)**	**Negative experience OR (95% CI)**	**Unwillingness to use OR (95% CI)**	**Reference to copper-related/systemic side effects OR (95% CI)**
**Income**				
High	Reference	Reference	Reference	Reference
Medium	Inconclusive	Inconclusive	Inconclusive	Inconclusive
Low	1.55 (1.12–2.15)	1.92 (1.03–3.57)	Inconclusive	Inconclusive
**Age**				
18–29	2.12 (1.55–2.90)	Inconclusive	2.38 (1.57–3.63)	Inconclusive
30–49	1.60 (1.21–2.12)	Inconclusive	2.90 (1.94–4.34)	Inconclusive
50–64	Reference	Reference	Reference	Reference

A low income was associated with negative attitudes toward (OR 1.55 [95% CI 1.12–2.15]) and negative experiences of the IUD (OR 1.92 [95% CI 1.03–3.57]), although no such association was found in relation to unwillingness toward future use or referring to systemic side effects. No associations were identified with educational level, country of birth, or parenthood. Younger age was associated with negative attitudes (OR 2.12 [95% CI 1.55–2.90]; OR 1.60 [95% CI 1.21–2.12]) and unwillingness toward future use of the IUD (OR 2.90 [95% CI 1.94–4.34]; OR 2.38 [95% CI 1.57–3.63]).

### Evaluation of Information and Healthcare

The respondents were asked about the accessing and evaluation of information or accounts about the copper IUD from different sources. They were asked to rate their level of satisfaction in healthcare in relation to the device, and also to assess their general confidence in several societal institutions (see Table 5).

**Table 5:** Associations between attitudes regarding information and healthcare and negative attitudes toward IUD, expressed as odds ratios (ORs) with 95% confidence intervals (CIs).

	**Negative attitude OR (95% CI)**	**Negative experience OR (95% CI)**	**Unwillingness to use OR (95% CI)**	**Reference to copper-related/systemic side effects OR (95% CI)**
**General reliability**				
Healthcare	1.64 (1.24–2.16)	2.42 (1.26–4.65)	inconclusive	2.29 (1.14–4.63)
Researchers	Inconclusive	2.42 (1.29–4.53)	1.62 (1.09–2.41)	2.56 (1.23–5.35)
Politicians	Inconclusive	1.93 (1.25–2.97)	Inconclusive	Inconclusive
School	1.39 (1.13–1.71)	1.91 (1.22–2.98)	1.35 (1.03–1.77)	Inconclusive
Social insurance agency	Inconclusive	Inconclusive	Inconclusive	1.97 (1.00–3.86)
Police	1.96 (1.54–2.50)	Inconclusive	1.52 (1.11–2.07)	Inconclusive
**Reliability on the copper IUD**				
Medical professionals	1.79 (1.31–2.43)	3.04 (1.41–6.54)	2.23 (1.54–3.23)	4.36 (2.27–8.40)
Regulatory institutions	1.63 (1.26–2.10)	2.90 (1.67–5.02)	2.03 (1.47–2.80)	3.16 (1.69–5.90)
CAM	0.67 (0.53–0.87)	Inconclusive	Inconclusive	0.32 (0.17–0.59)
Social media	0.54 (0.38–0.76)	Inconclusive	Inconclusive	0.31 (0.14–0.65)
Other people encountered in person	0.63 (0.52–0.78)	Inconclusive	0.68 (0.52–0.89)	0.40 (0.19–0.87)
Own experiences	0.59 (0.48–0.74)	Inconclusive	0.70 (0.54–0.92)	Inconclusive
				
**Having taken part in info/stories on risks associated with copper IUD**				
From healthcare	2.32 (1.75–3.09)	inconclusive	2.08 (1.42–3.04)	3.94 (2.08–7.48)
From CAM	4.20 (2.85–6.19)	2.33 (1.16–4.68)	2.61 (1.63–4.17)	3.12 (1.40–6.97)
From other individuals	3.23 (2.63–3.96)	inconclusive	1.78 (1.37–2.30)	4.35 (2.39–7.91)
Never	0.36 (0.30–0.44)	inconclusive	0.61 (0.48–0.78)	0.22 (0.11–0.43)
				
**Evaluation of information**				
Source criticism	inconclusive	inconclusive	inconclusive	inconclusive
Access to information	1.53 (1.22–1.92)	2.53 (1.66–3.86)	inconclusive	inconclusive
Easy to discern origin of information	1.61 (1.26–2.05)	2.79 (1.74–4.46)	1.51 (1.07–2.12)	2.43 (1.04–5.71)
Ability to assess quality, reliability	1.47 (1.16–1.87)	1.86 (1.17–2.96)	inconclusive	inconclusive
				
**Experiences with healthcare**				
Satisfied with healthcare	3.32 (2.11–5.24)	7.81 (3.37–18.13)	2.91 (1.60–5.30)	3.04 (1.23–7.51)
				
**Other attitudes**				
Vaccine hesitancy	inconclusive	inconclusive	1.51 (1.18–1.92)	inconclusive

Regarding the perceived reliability of information about the copper IUD, the respondents were asked to rate a number of sources, i.e., healthcare professionals; institutions regulating medical practice, such as the Swedish Medical Products Agency (Läkemedelsverket) and the National Board of Health and Welfare (Socialstyrelsen); complementary and alternative medicine (CAM) sources; social media; stories of other individuals whom the respondent had met; and one's own experiences on a four-point scale. Medical professionals were regarded as very or rather reliable by 91.0% of the participants, regulatory institutions by 85.6%, CAM sources by 15.0%, social media by 6.8%, stories of other individuals they had met in person by 65.9%, and one's own experiences by 70.0%. For the regression analyses, these variables were dichotomized into reliable vs. unreliable or unsure (*very reliable; rather reliable* vs. *rather unreliable; very unreliable; do not know*). An OR>1 indicates a lower evaluation of reliability than in the reference group, while an OR <1 shows a higher evaluation of reliability than in the reference group.

Healthcare professionals and regulatory institutions were rated as less reliable by all the groups at hand, i.e., by those with negative attitudes toward (OR 1.79 [95% 1.31–2.43]; OR 1.63 [95% CI 1.26–2.10]) or negative experiences of the IUD (OR 3.04 [95% CI 1.41–6.54]; (OR 2.90 [95% CI 1.67–5.02]), as well as by those unwilling to use an IUD in the future (OR 2.23 [95% 1.54–3.23]; OR 2.03 [95% CI 1.47–2.80]) and those referring to systemic side effects (OR 4.36 [95% CI 2.27–8.40]; OR 3.16 [95% CI 1.69–5.90]), compared to those not reporting negative attitudes, negative experiences, or unwillingness toward future use, nor referring to systemic side effects, respectively. The strongest presence of weak confidence was found among those with negative experiences of the IUD and among those referring to systemic side effects compared to those without such experiences and not making such references.

Complementary and alternative medicine sources and social media were rated as more reliable by those with a negative attitude toward the IUD (OR 0.67 [95% CI 0.53–0.87]; OR 0.54 [95% CI 0.38–0.76]) and by those referring to the systemic side effects (OR 0.32 [95% CI 0.17–0.59]; OR 0.31 [95% CI 0.14–0.65]), but not by those with negative experiences or those unwilling to use the device in the future. Other people's stories were rated as more reliable by those with a negative attitude (OR 0.63 [95% CI 0.52–0.78]), those unwilling toward future use of the IUD (OR 0.68 [95% CI 0.52–0.89]), and those referring to systemic side effects (OR 0.40 [95% CI 0.19–0.87]), but not by those with negative experiences. One's own experiences were regarded as more reliable by those with negative attitudes toward (OR 0.59 [95% CI 0.48–0.74]) or an unwillingness to use the IUD (OR 0.70 [95% CI 0.54–0.92]).

Questions were asked about whether respondents had taken a part of information or stories about risks pertaining to the copper IUD from healthcare professionals, CAM sources, or other individuals. A negative attitude toward the copper IUD, unwillingness to use the device, and referring to claimed systemic side effects were associated with having taken part of information from all these sources to a larger degree than those not reporting a negative attitude or an unwillingness toward future IUD use, and those not referring to unestablished systemic side effects. Negative experiences were associated with having taken part of the information from CAM actors, but not from other sources. Notably, due to the strength of the associations, those referring to systemic side effects had more commonly taken part of stories or information from other people (OR 4.35 [95% CI 2.39–7.91]) and from healthcare (OR 3.94 [95% CI 2.08–7.48]) compared to those not referring to such side effects, whereas those with a negative attitude toward the IUD had more commonly taken part in information from CAM sources (OR 4.20 [95% CI 2.85–6.19]) and from other people (OR 3.23 [95% CI 2.63–3.96]) compared to those reporting a positive attitude or being unsure.

Concerning the evaluation of information, self-assessed source criticism was measured through a question asking about the respondents' level of agreement with the statement, *it is generally important for me to be source critical and find out where health-related information comes from*, on a four-point scale. Additional questions inquired about whether respondents agreed that they had access to the information they needed about the copper IUD, and whether they found it easy to discern the origin and assess the quality and reliability of such information. The latter three questions enabled responses along a four-point scale, with an additional option that information had not been sought. The approximate one-third of respondents who chose that option were not included in the regression analyses.

Overall, strong agreement about the importance of source criticism was reported by 57.2%, while 38.7% reported being in fairly strong agreement. After excluding those reporting that they had not sought information, 40.2% were in strong agreement that they had access to sufficient information about the copper IUD, 30.4% that they found it easy to discern the origin of such information, and 31.1% that they had the ability to assess its quality and reliability. These variables were dichotomized for the logistic regressions (*agree strongly; agree fairly strongly* vs. *agree somewhat; disagree; unsure*).

None of the groups showed an association with agreeing less or being unsure about the importance of source criticism. Less agreement about having access to sufficient information was more common among those with negative attitudes toward (OR 1.53 [95% CI 1.22–1.92]) or experiences of (OR 2.53 [95% CI 1.66–3.86]) the copper IUD. Less agreement about finding it easy to discern the origin of information was more common among those with negative attitudes (OR 1.61 [95% CI 1.26–2.05]) or experiences (OR 2.79 [95% CI 1.74–4.46]), as well as among those reporting unwillingness to use an IUD in the future (OR 1.51 [95% CI 1.07–2.12]) and those referring to systemic side effects (OR 2.43 [95% CI 1.04–5.71]). Finally, less agreement about finding it easy to assess the quality and reliability of such information was more common among those with negative attitudes (OR 1.47 [95% CI 1.16-1.87]) or experiences (OR 1.86 [95% CI 1.17–2.96]), compared to those not reporting negative attitudes or experiences, respectively.

Regarding healthcare, 37.2% (*n* = 745) of the respondents reported having been in contact with medical personnel concerning the copper IUD. Of these, 86.5% (*n* = 644) were very (48.5%) or rather (38.0%) satisfied with those encounters. Being dissatisfied or unsure (*very satisfied; rather satisfied* vs. *rather dissatisfied; very dissatisfied; unsure*) was associated with a negative attitude toward (OR 3.32 [95% CI 2.11–5.24]) or an unwillingness toward future use of the IUD (OR 2.91 [95% CI 1.60–5.30]), as well as with referring to systemic side effects (OR 3.04 [95% CI 1.23–7.51]), and, most strongly, with negative experiences (OR 7.81 [95% CI 3.37–18.13]) of the IUD.

In addition, one of the survey questions inquired about the respondent's general attitudes toward vaccination. Regression analyses using the dichotomized version of this question (*very positive* vs. *less positive, negative, or unsure*) showed no conclusive association between vaccine hesitancy and negative attitudes or experiences of the IUD, or with referring to systemic side effects. Vaccine hesitancy was, however, associated with unwillingness toward future use of a copper IUD (OR 1.51 [95% CI 1.18–1.92]).

Finally, the participants rated their general confidence in healthcare, researchers, politicians, schools, the social insurance agency, and the police on a four-point scale (from *very strong* to *very weak*). Very or rather strong confidence was reported by 88.4% for healthcare, 90.6% for researchers, 36.2% in relation to politicians, 74.0% for schools, 38.8% for the social insurance agency, and 83.9% in the case of the police. The regression analyses were based on dichotomized versions of these variables, separating respondents reporting strong confidence from those reporting it to be weak (*very strong; rather strong* vs. *rather weak; very weak*).

A weaker confidence in healthcare was reported by those with negative attitudes toward (OR 1.64 [95% CI 1.24–2.16]) or negative experiences of the IUD (OR 2.42 [95% CI 1.26–4.65]) and by those referring to systemic side effects (OR 2.29 [95% CI 1.14–4.63]). A weaker confidence in researchers was reported by those with negative experiences of the IUD (OR 2.42 [95% CI 1.29–4.53]), those unwilling toward future use of an IUD (OR 1.62 [95% CI 1.09–2.41]), and those referring to systemic side effects (OR 2.56 [95% CI 1.23–5.35]). All the four groups also showed association with weaker confidence in some of the other societal institutions. The highest levels of weak confidence were found among those with negative experiences of the IUD and among those referring to systemic side effects.

### Open Text Responses

The first of the survey's two open questions about the copper IUD was a follow-up question posed to those (*n* = 329) responding that they did not want to use a copper IUD in the future, inquiring about the reason for this. The second one, targeting all respondents (*n* = 2000), invited any additional comments regarding the copper IUD. After the exclusion of non-indicative responses such as “no” and “no comment,” the first question yielded 312 and the second 338 responses. A total of 34 participants responded to both questions.

#### Comments Regarding Unwillingness to Use a Copper IUD

A large share of responses to the question regarding unwillingness toward future use of a copper IUD (Table 6) referred to *side effects*. Many of these (*n* = 81) mentioned the established and common side effects of increased menstrual bleeding and/or pain, as well as pain associated with IUD insertion. In a few cases, such side effects were described as quite severe, referring to pain as “constant” (R49), “incredible” (R99), or “crazy” (R206), and bleeding without interruption for 6 months (R229) or a year (R146). Many (*n* = 72) referred to unspecified side effects. One mentioned problems with having the IUD removed.

**Table 6:** Open survey responses regarding reported unwillingness to use a copper IUD: themes and categories.

**1. Why not copper IUD**		
Themes	Categories	Sub-categories
Side effects (*n =* 182)	Established/common (*n =* 81)	General (*n =* 74)
		Severe (*n =* 7)
	Unspecific (*n =* 72)	
	Other (*n =* 44)	Perforation, displacement (*n =* 12)
		Cysts, ectopic pregnancy, fertility, damage to uterus or ovaries (*n =* 6)
		Systemic/mental issues (*n =* 5)
		Copper/ metal (*n =* 22)
Other issues (*n =* 125)	Expulsion (*n =* 3)	
	Pregnancy (*n =* 4)	
	Endometriosis/PCOS (*n =* 4)	
	Foreign object (*n =* 54)	
	Feels unnatural (*n =* 5)	
	Men should also be responsible (*n =* 4)	
	Insufficient research/knowledge (*n =* 5)	
	Unspecific/Other (*n =* 39)	
Sources	Other people (*n =* 46)	
	Own experiences (*n =* 17)	
	Healthcare (*n =* 8)	
	Reading (*n =* 6)

I know several people who had difficult side effects of the copper IUD and who had trouble getting an appointment to take it out when the side effects didn't stop, which made it hard for them. (R282)

Other mentions of side effects (*n* = 44) included experiences of or worries about the IUD perforating body tissue, migrating, or getting stuck (*n* = 12), or other issues (*n* = 6) including cysts and ectopic pregnancy. Some referred to unestablished side effects of systemic character (*n* = 5) and/or negative effects of copper (*n* = 22), the latter in a few cases pointing to negative or allergic reactions to metals in general. A few of these (*n* = 3) used the concepts of excess copper or toxicity.

The copper is released into the body and affects the bodily balance negatively. (R201)

Some responses concerning side effects contained strong expressions, such as “nightmare/torture” (R99), “horror stories” (R34), and “terrible experiences” (R69).

The category gathering comments about *other issues* (*n* = 125) included references to IUD expulsion (*n* = 3), method failure through pregnancy (*n* = 4), incompatibility with endometriosis or PCOS (*n* = 4), not wanting a foreign object inside the body (*n* = 54), or the copper IUD feeling unnatural (*n* = 5). Some (*n* = 4) argued that men should also take active responsibility for contraception. A few referred to healthcare providing insufficient information, knowledge, or research (*n* = 5).

The risk of side effects is too big. Healthcare says there are hardly any, but I know several people who had a copper IUD and felt really bad during that time. (R292)

Comments also included general negative statements (*n* = 39) about the copper IUD or not wanting to use it.

Many responses provided no information about the *sources* on which the statements were based. Those who did referred to other people's stories (*n* = 46), personal experiences (*n* = 17), information or advice from healthcare (*n* = 8), or, unspecifically, to reading (*n* = 6). Social media were not explicitly mentioned.

#### Open Comments

Additional comments regarding the copper IUD (Table 7) were categorized based on the expressed *attitudes*, as positive (*n* = 53), value-neutral (*n* = 77), and ambivalent or negative (*n* = 208). Positive responses referred to personal experiences or more general attitudes or pieces of information.

**Table 7:** Open-survey responses regarding the copper IUD: themes and categories.

**2. Open question**			
Themes	Categories	Sub-categories	
Attitudes	Positive (*n =* 53)		
(*n =* 338)	Value-neutral (*n =* 77)		
	Negative/Ambivalent (*n =* 208)	Pros and cons (*n =* 44)	
		Established/common side	General (*n =* 61)
		effects (*n =* 96)	Severe (*n =* 35)
		Unspecific side effects (*n =* 33)	
		Other aspects (*n =* 54)	Pregnancy (*n =* 9); Expulsion (*n =* 6)
			Foreign object (*n =* 10); Unspecified negative (*n =* 16)
		Other side effects (*n =* 43)	Infections (*n =* 9); Perforation/dislocation (*n =* 13); Systemic/mental issues (*n =* 5); Copper/toxicity (*n =* 10)
Information,	Info/care worked well (*n =* 8)		
knowledge and healthcare (*n =* 67)	Problematic aspects (*n =* 58)	Individual experience (*n =* 22)	Info insufficient (*n =* 15); Discord info and personal experience (*n =* 5); On evaluation of info (*n =* 4)
		Wider issues (*n =* 41)	Lack of research, knowledge, support (*n =* 23); Information differences (*n =* 20); Uncertainty (*n =* 9)
		Importance of better info, practice in healthcare (*n =* 18)	
Sources	Own experience (*n =* 119); Healthcare (*n =* 50); Other people (*n =* 55); (Social) media (*n =* 5)

I've had it for more than 20 years, without any kinds of problems. (R45)

Among the ambivalent or negative comments (*n* = 208), many (*n* = 44) mentioned both pros and cons, noting, i.e., that risks and benefits must always be weighed against each other and that no contraceptive methods are perfect, at least not for all people. Some participants stated that they were positive toward the copper IUD despite having experienced some challenges with it. Many (*n* = 96) referred to common side effects, i.e., bleeding, menstrual pain, and pain on insertion. Around one-third of these (*n* = 35) described bleeding or pain as severe, and in some cases seriously interfering with daily life.

My bleedings became enormous and I couldn't live my life. It blead through [my menstrual protection] even when I just went to the grocery store. (R117)

Some (*n* = 33) referred to unspecific side effects. Others mentioned issues (*n* = 54) including method failure through pregnancy (*n* = 9), expulsion (*n* = 6), and feeling uncomfortable with inserting a foreign object (*n* = 10), in some cases with reference to metal (*n* = 2) in the body.

More general negative comments (*n* = 16) ranged from stating that better alternatives exist to declaring “never again!” (R197). Other mentioned side effects (*n* = 43) included infections (*n* = 9) and tissue perforation or IUD migration (*n* = 13), in some cases (*n* = 5) leading to surgery. Some comments pointed toward systemic side effects (*n* = 5), including effects on mood, hair loss, or “vague” symptoms. Others referred to effects attributed to copper (*n* = 10). The latter included references to copper toxicity.

I've thought about inserting one several times because of no hormones, but then I read about side effects which felt off-putting (e.g., copper intoxication). (R149)

There was little overlap between the two open survey questions regarding references to systemic side-effects. Thus, 42 respondents in total raised this theme.

Some negative comments included strong expressions such as references to “terrible” experiences (R93, R241, R254), “hell” (R83), “horror stories” (R95), and being “completely thrown off balance” (R159).

Many comments (*n* = 67) concerned *information, knowledge, or healthcare*. Of these, some (*n* = 8) spoke positively about information or healthcare having worked well, including in cases of experiencing side effects or problems. Others (*n* = 58) referred to problematic aspects of information, research, or healthcare at an individual level or as a wider issue. Among comments concerning the individual level (*n* = 22), several (*n* = 15) highlighted a perceived lack of information from healthcare providers about side effects or other aspects of, or alternatives to, the copper IUD. Some (*n* = 5) referred to a discord between information provided by healthcare and personal experiences related to the common side effects of heavy bleeding, to the less common one of tissue perforation, and to unestablished side effects.

I contacted healthcare after inserting an IUD because I bled and had pain. I was told that it's very uncommon that something goes wrong at insertion and that IUDs perforating the uterine wall was basically a myth, which was incorrect in my case. (R41)

Difficult to be taken seriously about side effects that came with the copper IUD, like loss of hair, strong pains etc. (R124)

Some respondents (*n* = 4) spoke about evaluating information about the IUD. Two of these expressed confidence in their evaluative ability due to medical training, while others referred to uncertainty and weighting information from different sources.

I mix medical knowledge … with friends' personal experiences, having taken part of things like how it feels to insert it, how it makes them feel etc. And then I weigh in my personal experiences. (R65)

With reference to wider issues (*n* = 39), some (*n* = 23) spoke of a lack of information, research, or support from the healthcare.

I think the scientific texts often lack perspectives on risks and problems that it can bring. Even if pregnancy is unwanted you don't want to risk your general health. There's a limit to how bad you can feel just for the sake of using contraception. (R338)

This category includes statements about a perceived need for more research on women's health issues (*n* = 2) and the development of contraceptive methods for men (*n* = 7).

Another noted issue (*n* = 20), also observed among responses to the first open question, was tension between different perspectives on copper IUD side effects. Some (*n* = 10) referred to a discrepancy between the information provided by healthcare and the experiences or stories of the users.

There's a big discrepancy between the side effects indicated by healthcare for the copper IUD and those that people who have experienced side effects speak about. When they appear they're often serious, it seems. (R192)

I think generally there's far too much information from healthcare and authorities about the benefits of contraceptives and far too little on side effects and risks. You hear about side effects from personal contacts. I have strong confidence in healthcare overall, but relatively low confidence when it comes to this particular issue. (R285)

There are many of us who aren't believed by healthcare, how we've felt after a few years with a copper IUD. “You can't feel that way because of a copper IUD”. (R200)

A few (*n* = 3) referred to commercial interests potentially or likely affecting information about the IUD.

A sense of uncertainty regarding information about the copper IUD was expressed (*n* = 9), relating related both to accounts shared via social contacts and to existing medical knowledge or research on side effects.

I've heard many good things from healthcare staff, but almost only bad things from friends who have used it. I know, though, that it's usually only the ones with problems who share information with others. (R10)

I still wonder about other not yet established potential side effects. (R41)

I think the problem here is that it can be rather “fuzzy” side effects that aren't always easy to connect to the copper IUD. But there aren't many better options, especially not that suit everyone. (R316)

Among such comments addressing uncertainty, some (*n* = 4) referred to effects of copper or copper toxicity, and to information shared via social media.

A lot of rumors about copper intoxication that healthcare staff can't respond to. (R33)

Relatedly, some (*n* = 18) referred to the importance of improved communication, provision of information, and practice in healthcare.

In my view, there's a need for more research on copper IUDs. (R139)

Clearer info about risks and more research should be required. For me the copper IUD triggered endometriosis that I have to live with for the rest of my life. (R299)

I think it's important that healthcare gives a more nuanced picture when recommending a copper IUD. (R56)

One comment invoked a historical perspective while stating that women's experiences should be taken seriously and simultaneously posing a question about the effects of copper.

Invest more in research concerning women's bodies overall. What effects do high levels of copper have if they are released in a woman's body? Don't diminish women's experiences or side effects, as if that's something that should be fine or manageable for women, oh how women have had to suffer through history. Listen when women speak! (R322)

Regarding healthcare practice, one comment referred to the common breakage of one type of IUD and was critical about users not having been contacted for examination. Moreover, a couple of responses referred to the risk of lacking information or support from healthcare creating an opening to other sources of information, i.e., “Google” (R48) or alternative health actors.

There's a lack of sufficiently reliable and updated information about side effects and risks that have emerged after the IUD was approved. There's also a lack of interest in and knowledge about the risks of heightened levels of systemic copper, i.e., that is taken up by the bloodstream and affect the rest of the body. That creates an opening, unfortunately, for amateurish opinion-makers, quackers and influencers. Here healthcare and pharmaceutical companies should show more interest and take their responsibility. (R240)

It hurt terribly to insert it, I menstruated strongly 12 days a month. I constantly got urinary tract and fungal infections during the year I had the copper IUD, after taking it out in 2017 I haven't had that a single time. I would have wanted information before about for how long you should give it a chance and what to do if it doesn't work for me. I felt abandoned after having it inserted. I would have wanted a follow-up call from healthcare, so that I'm not just left with Google. (R48)

Of those who referred to any *sources* on which their comments were based, most (*n* = 119) pointed to personal experiences of copper, or in a couple of cases hormonal, IUDs. Many (*n* = 50) referred to information or advice, or lack thereof, from healthcare or research, while others (*n* = 55) spoke of experiences or stories of other people, such as family members or friends. A few (*n* = 5) referred to (social) media. Of the four respondents mentioning social media, one reported finding it helpful to read about other people's experiences, one stated she was surprised that some people believe what they read in social media, and two said social media had increased their insecurity or disinclination toward the copper IUD.

I think this is a bit harder to relate to than many other (medical) debates, given the number of stories one has taken part of in social media. (R103)

## Discussion

This survey study assessed attitudes toward the copper IUD among adult women in Sweden. While many reported positive experiences and attitudes, just over one-third (34.7%) reported negative attitudes through indicating weak or no agreement with one or more of the statements that the side effects of the IUD are mild and uncommon, that the benefits of the copper IUD outweigh its potential risks, and that it fulfills an important function in preventing unwanted pregnancy. Almost half (45.4%) of the present or former copper IUD users reported negative experiences. Of those without past or present experience of copper IUD use, one in four (25.2%) reported not wanting to use a copper IUD in the future, choosing this option over other ones including those indicating no need for a contraceptive or preference for another contraceptive method.

Moreover, many of the open survey responses expressed ambivalent or negative attitudes. Responses to the first open survey question, regarding the reported unwillingness to use a copper IUD, would be expected to be negative due to the nature of the question. Several responses to the second open question included references to both positive and negative aspects of the copper IUD or comparisons with other contraceptive methods, on occasion noting that no contraceptive method is entirely without problems (14, 18). Nevertheless, negatively oriented comments had a strong presence among the open responses, overall. This is in correspondence with previous research noting negative attitudes toward the copper IUD (14, 16, 18, 20, 21), although positive experiences and attitudes have also been shown (6, 14, 15, 23).

Regarding sociodemographic variables, no associations were identified with educational level, country of birth, or parenthood. Low income was associated with negative attitudes toward and experiences of the copper IUD, while low or medium age was associated with negative attitudes toward and unwillingness toward future use of the copper IUD. While these associations are worth noting, the results indicate that negative attitudes toward or experiences of the copper IUD or making references to systemic side effects are not strongly determined by sociodemographic factors.

### Side Effects and Other Negative Events

Among the respondents, one in four reported agreeing to a fairly low degree or not at all that the side effects of the copper IUD are uncommon (25.8%) or mild (25.5%). Many open responses referred to established and common or unspecific side effects, although in some cases expressing a stronger severity of symptoms than what may perhaps be expected (48). In line with findings from other studies (14, 16, 48), other negatively oriented responses included references to method failure through pregnancy, expulsion, discomfort with inserting a foreign object inside the body, infections, tissue perforation or IUD migration, and triggering or being incompatible with endometriosis.

In addition, 42 (2.1%) of the open responses referred to unestablished systemic side effects of the copper IUD. While these responses come from a small share of respondents, they are notable as this type of perceived side effects was not inquired about or otherwise mentioned in the survey or the accompanying text. This result suggests, therefore, that notions of such side effects are not entirely uncommon in the Swedish population.

In open survey responses, references to side effects, sometimes described in strong terms or as being severe, were articulated alongside comments about having received insufficient information about such side effects from healthcare providers.

### The Use and Perceived Reliability of Information

Previous findings that social networks have importance for choices of and attitudes toward contraceptives (16, 22, 28, 29, 70), that women often use a combination of sources of information about the copper IUD (16, 22), and that some women consider accounts about the IUD provided via social contacts to be more reliable than information provided by healthcare professionals (22, 30, 32) find some support in the present study. Those with negative attitudes and unwillingness toward future use of the copper IUD, as well as those referring to systemic side effects, reported having taken part of the information about risks associated with the IUD from healthcare providers as well as from CAM sources and other individuals to a larger degree than those not reporting negative attitudes or experiences or mentioning unestablished side effects. Meanwhile, social media and other people encountered in person, as well as CAM sources were regarded as more reliable by those with negative attitudes, those referring to unestablished side effects, and, in the case of other people encountered in person, by those unwilling toward future use of a copper IUD. In addition, references to other people's experiences were strongly present in the open survey responses, as were accounts of personal experiences. One survey response spoke clearly about using and combining information from different sources. The latter is in line with the findings of our own qualitative study of women making claims about unestablished systemic or copper-related side effects of the copper IUD (17).

Moreover, while many studies on attitudes toward IUDs emphasize the importance of countering negative stories, expressed via social networks not least online, through countering misinformation (22, 30, 32) and relaying positive experiences of the IUD (22, 70), several survey respondents called for more or improved information, including updated research from healthcare professionals (17).

A motivation for emphasizing the importance of communicating positive information about the IUD is the observation that negative stories or experiences tend to be regarded as more noteworthy and memorable and have a greater effect on prospective user attitudes than positive ones (22, 70). While this is likely true, IUD discontinuation has been associated with not feeling sufficiently informed about potential side effects (14, 77), with feeling that experienced side effects were minimized by healthcare providers, and not feeling listened to or respected in the clinical encounter, for example, when requesting IUD removal (78). Similar processes have been noted in relation to other contraceptives (79). Meanwhile, Holt et al. (47) found that while women wanted clear, complete, and accurate information about contraceptive methods, a fundamental precondition for communication of such information was the establishment of a relationship of trust. Mistrust in and concerns with the quality of healthcare have, moreover, not only been tied to resistance to or discontinuation of IUD use (14) but also to the spread of negatively charged or incorrect health-related information online (34).

It is, in this light, notable that negative attitudes toward and experiences of the IUD, as well as making references to unestablished side effects were associated with a weaker confidence in institutions regulating medical practice and with healthcare providers both specifically regarding the copper IUD (and with the exception of unwillingness toward future use of an IUD) in general. All four groups also showed association with a lower level of satisfaction with healthcare in relation to the copper IUD. The association with such dissatisfaction, as well as with lower levels of confidence in healthcare was notably strong among those with negative experiences of the copper IUD.

Probable reasons behind the reported lack of confidence in the information provided by healthcare providers includes the contemporary increase in the contestation of established expertise (35), in a context where levels of mis/trust in societal institutions including medical ones exist in interplay with each another (44). Worth noting here, and in line with a previous study (22), some survey responses referred to healthcare professionals as a source of positive information about the IUD, whereas negative information or information about side effects was reported to be shared via social contacts. As mentioned above, Anderson et al. (22) point out that stories with negative content were seen by some women as not only more prevalent but more memorable than positive ones, similarly to a greater online popularity of negative accounts, compared to positive ones, concerning other medical interventions (34). In the case of the copper IUD, this can presumably be related to established contraceptive methods being assumed to work well, meaning that unexpected adverse events are more noteworthy (22). The notion that information about side-effects is primarily communicated via social contacts can also, however, be set in relation to research observing tendencies toward omitting discussion or downplaying the existence of side effects of contraceptives on the part of healthcare professionals (80, 81).

Moreover, the expressed sense of discord between information from healthcare providers and personal or other people's related experiences is significant not least in relation to research showing an association between the presence of conflicting stories and a sense of ambiguity regarding evidence, which is conducive to the spread of rumors and negatively charged accounts online (34). Accordingly, it has been observed (22) that being recommended an IUD by a healthcare professional but hearing from someone else that it could cause serious harm might impact trust in clinicians. This may be one aspect of the lower confidence in healthcare providers and agencies regulating medical practice reported by respondents with negative experiences or attitudes of the copper IUD. Meanwhile, some open survey responses acknowledged the uncertainty or potential unrepresentativity of negative comments accessed through social networks (16). Worth noting here is that a lower evaluation of the importance of source criticism was not associated with reporting negative attitudes or experiences, or referring to unestablished side effects, compared to those who did not, although this is not necessarily a good measure of actual source criticism. In any case, this underscores the importance of healthcare providers providing clear information on and being willing to discuss women's concerns about the copper IUD, including its potential side effects, in the interest of promoting trust.

All four groups reporting negative attitudes or experiences, or making references to unestablished side effects, had taken part of information about copper IUD from CAM sources to a larger degree than respondents not reporting negative attitudes or experiences and not referring to such side effects. Moreover, CAM sources as well as social media and other people's stories were regarded as more reliable by those with negative attitudes and those referring to unestablished side effects. Notably, a few open survey responses stated that a perceived lack of information from healthcare can provide a motivation for searching for input or support from other sources. This is in line with Larson et al.'s (44) argument that when trust in medical and other societal institutions is weakened, confidence may instead be placed in other sources, something that is also supported by our qualitative research on women claiming to have experienced unestablished side effects of the copper IUD, who turned to CAM sources due to feeling that healthcare did or could not provide help (17). This may be an aspect of the association between negative experiences of the copper IUD and taking part of the information from CAM sources.

With reference to trust in medical institutions, Larson et al. (44) emphasize the importance of past experiences. Relatedly, the current tendency toward lack of trust in science and medicine has been related to historical and contemporary structures of inequality and injustice (40, 82), i.e., to historical or personal experiences of being deprioritized or maltreated by institutions, including healthcare (83). History offers several examples of negative effects of contraceptive methods and their methods of administration, which have initially gone unrecognized by medical authorities (51–57, 61). Worth mentioning here is that the regulation of medical devices, among which copper IUDs are included, has been criticized for being less rigorous than that of pharmaceutical drugs (84, 85). Further, research has also pointed to women feeling uninformed, unheard, or dismissed by healthcare providers in relation to contraceptives and their (potential) side effects (31, 60, 62–64). Examples of the latter appear among the open survey responses, referring to experiences of finding it difficult to get the copper IUD removed on request (62, 77, 86), and of not being taken seriously by healthcare providers when mentioning perceived side effects (17). Such comments are concerning from the perspective of reproductive autonomy and rights (55, 77), which include access to healthcare supporting the right to make decisions about one's own body, free from stigma or coercion (87, 88), and the diagnosis and treatment of unhealthful effects of contraceptives, and which should be characterized by a women's rights perspective at every level (89). Such responses are also concerning due to potential implications for the continued clinician–patient relationship, for future contraceptive use (16, 77, 90) and for future trust in healthcare.

In sum, while this study supports the importance of healthcare providers communicating correct information and countering misunderstandings about the IUD (11, 14, 27), it also affirms the importance of healthcare providers promoting trust by providing clear information about potential side effects (14) and being open to respectfully discuss women's experiences and concerns (33, 49, 81). As noted by Hoggart and Newton (62), although providing full information about potential side effects of contraceptive methods while not alarming prospective users unnecessarily may require a difficult balancing act, the failure to discuss side effects which are then experienced can lead to distrust of the healthcare provider.

A perhaps particularly difficult balancing act is demanded by healthcare providers when it comes to addressing women's claims about unestablished systemic side effects of the copper IUD. While health-related misinformation is a serious problem, in line with research emphasizing the continuous development of medical expertise and practice (91, 92) and historical records of previously unacknowledged side effects subsequently gaining official recognition (57, 61), we stress the need for moving beyond simple framings of accounts of unestablished side effects of the kind reported in this study as always and necessarily being a result of misinformation.

### Limitations

While this study was distributed via a web panel gathering randomly selected participants representative of people living in Sweden with regular access to internet, the response rate was limited to 37%. It is not unlikely that those with a negative attitude toward the copper IUD were more inclined to participate. This is however an issue common to most survey studies and the response rate was similar to other studies conducted by Kantar Sifo. In addition, the survey also addressed HPV vaccination, which decreases the risk of self-selection of women with a particular interest in the copper IUD. The share of respondents aged 18–49 years reporting current copper IUD use was 4.7%, which was however a bit lower than the share of women aged 15–49 years who used it during 2010–2013 (7%) (13), since when the share of women using LARCs increased (11). Our results were weighted using national weights pertaining to age and region provided by Statistics Sweden. Meanwhile, our results have limited generalizability to other contexts, such as countries in the Global South. Furthermore, while it would be interesting to compare attitudes toward copper IUDs with perceptions of hormonal IUDs, not least due to evidence suggesting some overlap between negative attitudes toward both types of devices (21), this study does not allow for that.

### Conclusion and Practice Implications

While positive experiences of and attitudes toward the copper IUD were expressed by the study participants, negative ones were also common. Our results suggest that there is room for improvement in healthcare provider communication about the copper IUD, including the promotion of trust by providing clear information about potential side effects (14) and being open to respectfully discuss women's experiences and concerns (49), while supporting women in their contraceptive choices (77, 78). Further research into reasons for IUD dissatisfaction, and ways in which health professionals do and may best respond to it, is warranted.

## Data Availability Statement

The raw data supporting the conclusions of this article are available via the corresponding author, upon reasonable request.

## Ethics Statement

The study involved human participants and was reviewed and approved by Swedish Ethical Review Authority (no. 2019-03017). Informed consent for participation was provided through the completion of the survey and submission of responses.

## Author Contributions

MW coordinated the design of the study, the analysis and interpretation of results, and the writing and revision of the manuscript. LG contributed to the design of the study, to the interpretation of results, and to the writing and revision of the manuscript. Both authors have approved the final version of the manuscript.

## Funding

This research was supported by FORTE—Swedish Research Council for Health, Working Life and Welfare (Dnr 2018-00951).

## Conflict of Interest

The authors declare that the research was conducted in the absence of any commercial or financial relationships that could be construed as a potential conflict of interest.

## Publisher's Note

All claims expressed in this article are solely those of the authors and do not necessarily represent those of their affiliated organizations, or those of the publisher, the editors and the reviewers. Any product that may be evaluated in this article, or claim that may be made by its manufacturer, is not guaranteed or endorsed by the publisher.
