# Supplementary material for: Attitudes Toward the Copper IUD in Sweden: A Survey Study
Source: Front Glob Womens Health. 2022 Jul 8;3:920298. doi: 10.3389/fgwh.2022.920298 (PMC9304811; doi:10.3389/fgwh.2022.920298)
Supplement: Supplementary file 1 [file Data_Sheet_1.PDF]

Kvinnors uppfattningar om kopparspiral och HPV-vaccin

Välkommen till en mätning från SIFO.

Vi är intresserade av att höra just DIN åsikt om följande frågor. Tänk på att det inte finns något rätt eller fel svar.

Vi är intresserade av dina spontana svar på frågorna, så fastna alltså inte alltför länge på någon fråga.

Denna undersökning genomförs på uppdrag av Lunds Universitet. Mer information om studien hittar du här.

Tryck på knappen nedan för att komma till första frågan.

Denna undersökning vänder sig till kvinnor. För att försäkra oss om att du ingår i målgruppen frågar vi dig: Vilken är din könsidentitet?

- Kvinna
- Man
- Annan könsidentitet

Vad har du för högsta avslutade utbildningsnivå?

- Grundskola (el. motsvarande)
- Gymnasium (el. motsvarande)
- Högskola, universitet eller annan eftergymnasial utbildning (ex. folkhögskola)
- Forskarutbildning
- Saknar fullföljd utbildning
- Vill ej svara
- Tveksam, vet ej

Var är du själv, din mor och din far födda? I Sverige eller i något annat land?

	Född i Sverige	Född i övriga Norden	Född i övriga Europa	Född utanför Europa	Vill ej svara
Jag själv					
Min mor					
Min far					

Har du några barn, oavsett hur gamla de är?

- Ja, som bor hos mig
- Ja, som bor växelvis hos mig
- Ja, som inte bor hos mig
- Nej, jag har inga barn
- Vill ej svara

Hur stor är din personliga månadsinkomst?
Med inkomst menar vi inkomst före skatt. Studiemedel, hyresbidrag, pensioner mm räknas också som inkomst.

- Mindre än 13 999 kronor/månad
- 14 000 - 24 999 kronor/månad
- 25 000 - 41 999 kronor/månad
- Mer än 41 999 kronor/månad
- Tveksam, vet ej
- Vill ej svara

Hur stort eller litet förtroende har du för följande institutioner/grupper i samhället?

	Mycket stort förtroende	Ganska stort förtroende	Ganska litet förtroende	Mycket litet förtroende	Tveksam, vet ej	Vill ej svara
Sjukvården						
Skolan						
Polisen						
Försäkringskassan						
Politiker						
Forskare						

Vad har du för inställning till vaccin i allmänhet?

- Mycket positiv
- Ganska positiv
- Ganska negativ
- Mycket negativ
- Tveksam, vet ej
- Vill ej svara

I vilken mån håller du med om följande påstående?
Det är generellt sett viktigt för mig att vara källkritisk och ta reda på var hälsorelaterad information kommer ifrån

- Instämmer i mycket hög grad/helt och hållet
- Instämmer i ganska hög grad
- Instämmer i ganska låg grad
- Instämmer i mycket låg grad/inte alls
- Tveksam, vet ej
- Vill ej svara

Kopparspiral

Kopparspiral används som ett preventivmedel för att förebygga oönskade graviditeter.

Har du eller har du haft kopparspiral?

- Ja, jag har kopparspiral
- Ja, jag har haft kopparspiral
- Nej, jag har inte haft kopparspiral
- Tveksam, vet ej
- Vill ej svara

Om Ja, jag har eller har haft kopparspiral:

Har du positiva eller negativa erfarenheter av att använda kopparspiral?

- Mycket positiva
- Ganska positiva
- Ganska negativa
- Mycket negativa
- Tveksam, vet ej
- Vill ej svara

Om Nej, jag har inte haft kopparspiral:

Tror du att du kommer att använda kopparspiral i framtiden?

- Nej, eftersom jag inte behöver något preventivmedel
- Nej, eftersom föredrar ett annat preventivmedel
- Nej, eftersom jag inte vill använda kopparspiral
- Ja, det är sannolikt
- Tveksam, vet ej
- Vill ej svara

Om Nej, eftersom jag inte vill använda kopparspiral:

Du uppgav att du inte vill använda kopparspiral. Berätta gärna kort om orsaken till detta.

Om du tänker på kvinnor i allmänhet, hur väl instämmer du i följande påståenden?

	Instämmer i hög grad/helt och hållet	Instämmer i ganska hög grad	Instämmer i ganska låg grad	Instämmer i mycket låg grad/inte alls	Tveksam, vet ej	Vill ej svara
Kopparspiralen fyller en viktig funktion för att förhindra oönskad graviditet						
Kopparspiralens risker och biverkningar är ovanliga						
Kopparspiralens risker och biverkningar är milda						
Kopparspiralens nytta överväger dess risker						

Har du varit i kontakt med vårdpersonal om kopparspiral?
Med kontakt menar vi att du antingen kontaktat vården enbart i syfte att prata om kopparspiral, eller sätta in kopparspiral alternativt att kopparspiral kommit på tal i samband med annat vårdärende.

- Ja
- Nej
- Tveksam, vet ej
- Vill ej svara

Om Ja:

I vilken grad är du nöjd med vårdpersonalens bemötande när du pratat med dem om kopparspiral?

- Mycket nöjd
- Ganska nöjd
- Ganska missnöjd
- Mycket missnöjd
- Tveksam, vet ej
- Vill ej svara

I vilken grad bedömer du att följande informationskällor är pålitliga eller opålitliga gällande kopparspiral?

	Mycket pålitlig	Ganska pålitlig	Ganska opålitlig	Mycket opålitlig	Tveksam, vet ej	Vill ej svara
Barnmorska, läkare eller annan vårdpersonal (inkl 1177, vård via digitala appar från legitimerad vårdpersonal)						
Läkemedelsverket, Socialstyrelsen eller annan offentlig (vård)institution (utöver läkare och annan vårdpersonal)						
Alternativ- eller komplementärmedicinska utövare eller texter						
Facebook, Youtube, bloggar eller andra sociala medier						
Personer du mött, som berättat om sina erfarenheter eller perspektiv						
Egna erfarenheter						

I vilken mån håller du med om följande påståenden?

	Instämmer i mycket hög grad/helt och hållet	Instämmer i ganska hög grad	Instämmer i ganska låg grad	Instämmer i mycket låg grad/inte alls	Tveksam, vet ej	Jag har inte behövt eller eftersökt information om kopparspiral	Vill ej svara
Jag har haft tillgång till den information jag behövt om kopparspiral							
Jag tycker att det är lätt att urskilja var information om kopparspiral kommer ifrån							
Jag har förmåga att bedöma kvaliteten och tillförlitligheten hos olika sorters information om kopparspiral							

Har du tagit del av information eller berättelser om risker förknippade med kopparspiral via sociala medier (t ex Facebook, youtube eller bloggar)?
Du kan markera fler än ett svarsalternativ

- Ja, information från sjukvård eller annan offentlig vårdinstutition
- Ja, information från alternativ- eller komplementärmedicinsk källa
- Ja, privatpersoners erfarenheter eller perspektiv
- Nej, aldrig
- Tveksam, vet ej
- Vill ej svara

Vill du tillägga något om kopparspiralen?
Utifrån egna erfarenheter eller mer generella tankar.
